# Comparability of accelerometer signal aggregation metrics across placements and dominant wrist cut points for the assessment of physical activity in adults

**DOI:** 10.1038/s41598-019-54267-y

**Published:** 2019-12-03

**Authors:** Jairo H. Migueles, Cristina Cadenas-Sanchez, Alex V. Rowlands, Pontus Henriksson, Eric J. Shiroma, Francisco M. Acosta, Maria Rodriguez-Ayllon, Irene Esteban-Cornejo, Abel Plaza-Florido, Jose J. Gil-Cosano, Ulf Ekelund, Vincent T. van Hees, Francisco B. Ortega

**Affiliations:** 1 PROFITH “PROmoting FITness and Health through physical activity” Research Group, Sport and Health University Research Institute (iMUDS), Department of Physical Education and Sports, Faculty of Sport Sciences, University of Granada, Ctra. Alfacar s/n, 18011 Granada, Spain; 2 Diabetes Research Centre, University of Leicester, Leicester General Hospital, Leicester, UK; 3 NIHR Leicester Biomedical Research Centre, Leicester, UK; 4 Alliance for Research in Exercise, Nutrition and Activity (ARENA), Sansom Institute for Health Research, Division of Health Sciences, University of South Australia, Adelaide, Australia; 5 Department of Biosciences and Nutrition, Karolinska Institutet, Huddinge, Sweden; 6 Laboratory of Epidemiology and Population Science, National Institute on Aging, Bethesda, MD USA; 7 Center for Cognitive and Brain Health, Department of Psychology, Northeastern University, Boston, MA USA; 8 Department of Sport Medicine, Norwegian School of Sport Sciences, Oslo, Norway; 9 Netherlands eScience Center, Amsterdam, The Netherlands; 10 Department of Medical and Health Sciences, Linköping University, Linköping, Sweden

**Keywords:** Occupational health, Epidemiology

## Abstract

Large epidemiological studies that use accelerometers for physical behavior and sleep assessment differ in the location of the accelerometer attachment and the signal aggregation metric chosen. This study aimed to assess the comparability of acceleration metrics between commonly-used body-attachment locations for 24 hours, waking and sleeping hours, and to test comparability of PA cut points between dominant and non-dominant wrist. Forty-five young adults (23 women, 18–41 years) were included and GT3X + accelerometers (ActiGraph, Pensacola, FL, USA) were placed on their right hip, dominant, and non-dominant wrist for 7 days. We derived Euclidean Norm Minus One *g* (ENMO), Low-pass filtered ENMO (LFENMO), Mean Amplitude Deviation (MAD) and ActiGraph activity counts over 5-second epochs from the raw accelerations. Metric values were compared using a correlation analysis, and by plotting the differences by time of the day. Cut points for the dominant wrist were derived using Lin’s concordance correlation coefficient optimization in a grid of possible thresholds, using the non-dominant wrist estimates as reference. They were cross-validated in a separate sample (N = 36, 10 women, 22–30 years). Shared variances between pairs of acceleration metrics varied across sites and metric pairs (range in r^2^: 0.19–0.97, all p < 0.01), suggesting that some sites and metrics are associated, and others are not. We observed higher metric values in dominant vs. non-dominant wrist, thus, we developed cut points for dominant wrist based on ENMO to classify sedentary time (<50 m*g*), light PA (50–110 m*g*), moderate PA (110–440 m*g*) and vigorous PA (≥440 m*g*). Our findings suggest differences between dominant and non-dominant wrist, and we proposed new cut points to attenuate these differences. ENMO and LFENMO were the most similar metrics, and they showed good comparability with MAD. However, counts were not comparable with ENMO, LFENMO and MAD.

## Introduction

Physical behaviors occurring during the 24 hours of the day consist of physical activity (PA), sedentary behavior and sleep. These three behaviors are of major public health interest due to their well-documented influence on health^1–4^. Objective methods to assess free-living PA range from cost-efficient tools such as pedometers to relatively expensive multi-sensor devices, e.g., Actiheart^5^. Accelerometers provide a balance of cost and feasibility and have been increasingly used in large epidemiological cohorts^6,7^, for example in the Women’s Health Study (WHS) and the National Health And Nutrition Examination Survey in the United States (NHANES), the Biobank study in the United Kingdom (UK Biobank), and the International Study of Childhood Obesity, Lifestyle and the Environment (ISCOLE) which collected data worldwide. Furthermore, accelerometers have been validated for the estimation of PA^8–10^, sedentary time^11–13^ and sleep^14–16^. However, accelerometer utilization requires data collection and processing decisions which could affect the final estimations^17^.

Data collection decisions start with the selection of the most appropriate anatomical location to attach the accelerometer^17^. Hip and wrist are the most frequently selected locations^17^, and both have been demonstrated to be valid locations for classifying PA intensities and sedentary time^8–10,17^, as well as to assess sleep^14–16^. However, it is unclear how much the accelerometer outcome measures vary between body sites. Previous studies have found high correlations (i.e., intraclass-correlation coefficients >0.9) between acceleration values from both wrists^18^ with slightly lower values in the non-dominant wrist, although non-significantly different from the dominant wrist^19,20^. Likewise, moderate-to-high correlations between acceleration values from either wrist and the hip have been reported (i.e., r coefficients between 0.7 and 0.9)^20,21^. These studies had a focus on PA and sedentary time (i.e., waking hours) and did not report associations during sleeping hours. Furthermore, cut points to estimate PA have been proposed for the non-dominant wrist^8,11^ and hip^8,11,22^ in adults, but not for the dominant wrist. Therefore, studies where accelerometers were placed on the dominant wrist do not have specific cut points proposed for their data, for instance the UK Biobank trial^23^.

Data processing aims to remove the gravitational component and noise from the raw signal, in order to obtain an acceleration signal aggregation metric (from herein acceleration metric) intended to reflect body movement^24^. For example, acceleration metrics include Euclidean Norm Minus One *g* with negative values rounded to zero (ENMO)^24^, ENMO of the low-pass filtered raw accelerations (LFENMO)^23^, Mean Amplitude Deviation (MAD)^25,26^ as well as manufacturer-specific metrics such as ‘activity counts’ (hereinafter counts), among others (see definitions of these acceleration metrics in Table 1). To our knowledge, these acceleration metrics have not previously been compared to each other in the same study. Comparing these metrics using data from hip, dominant wrist and non-dominant wrist and focusing on different periods of the day (i.e., 24 hours, waking and sleeping hours) could benefit researchers interested in either PA, sedentary time and/or sleep.

**Table 1:** Brief description of the acceleration metrics included.

Acceleration metric	Frequency filter	Definition
ENMO	None	Euclidean norm minus one *g* of the raw accelerations, with resulting negative values rounded to zero and then averaged over 5 s epochs.
LFENMO	Low-pass	Euclidean norm minus one *g* of the low-pass filtered raw accelerations with resulting negative values rounded to zero (Butterworth 4^th^ order filter; ω = 20 Hz).
MAD	None	Euclidean norm of each raw acceleration data point minus the mean of its correspondent 5 s epoch.
Counts	Band-pass	Counts are obtained by using a band-pass frequency filter to the raw signal (by default: ω_0_ = 0.025 Hz, ω_1_ = 2.5 Hz). The rest of information is mostly unknown.

ENMO: Euclidean norm minus 1 *g*; LFENMO: Low-pass filtered ENMO; MAD: Mean amplitude deviation.

Moreover, the movement pattern identified throughout the day by each of these metrics (i.e., acceleration metric values throughout the day) and the data from different body attachment sites may be useful to describe PA^27^. Only one study has investigated differences in movement patterns identified from hip, dominant wrist and non-dominant wrist^19^. This study only analyzed counts during waking hours in a sample of older adults^19^. The drawbacks of using brand-dependent activity counts have been described (e.g., precludes comparison across studies, complicates the interpretation of results, summarizes raw data which may minimize their potential)^28^, as well as the importance of moving forward with open-source derived metrics from raw accelerometer data^29,30^. Therefore, the present study aimed to: 1) study the comparability between different acceleration metrics across right hip, dominant wrist and non-dominant wrist attachment sites during different periods of the day (i.e., 24 hours, waking and sleeping hours); and 2) use previously established cut points for accelerations measured at the non-dominant wrist^8,11^ to develop and cross-validate cut points in a separate sample for accelerations measured at the dominant wrist in a sample of young adults.

## Methods

### Study design and participants

The present cross-sectional study analyzed free-living data from a convenience sample composed of students and research personnel from the University of Granada, Spain. The study was carried out in two waves of 45 (23 women, 18–41 years old) and 36 (10 women, 22–30 years old) young adults, respectively. Wave 1 (cut-point calibration sample) was used to compare different acceleration metrics across body attachment sites and to develop cut points for the dominant wrist that are consistent with the only set of cut points proposed to estimate PA intensity from the non-dominant wrist in adults to date^8,11^. Wave 2 (cut-point cross-validation sample) data were used to cross-validate the new set of cut points in a different sample of participants with similar characteristics. All participants were informed of the purpose of the study and written informed consent was obtained. This study was conducted according to the Declaration of Helsinki and approved by the Ethics Committee on Human Research (CEIH) of the University of Granada and the study was approved by the institutional review board of the University of Pittsburgh and National Institute on Aging.

### Procedures

Participants’ body weight and height were measured to the nearest 0.1 kg and 0.1 cm using an electronic scale (SECA 861, Hamburg, Germany) and a precision stadiometer (SECA 225, Hamburg, Germany). We calculated body mass index (BMI) as mass (kg)/height^2^ (m^2^). Participants were instructed to wear accelerometers (ActiGraph GT3X+, Pensacola, FL, USA) for seven complete days (24 hours per day). Cut-point calibration sample participants wore three accelerometers placed on the right hip, dominant wrist and non-dominant wrist. Cut-point cross-validation sample participants wore accelerometers on both wrists. All participants were instructed to remove the accelerometers during bathing and showering, to always wear and remove all of the accelerometers at the same time, and to keep a diary of the times they went to bed and got off the bed every day.

### Accelerometers

ActiGraph GT3X+ is a triaxial accelerometer with a dynamic range of +/− 6 G. Accelerometers were initialized to capture and store accelerations at 100 Hz. Raw accelerations were then downloaded (“.gt3x” files) and converted to “.csv” format using ActiLife v.6.13.3 (ActiGraph, Pensacola, FL, USA).

### Data processing

Raw “.gt3x” files were loaded in the ActiLife software to export raw data in.csv format and to obtain counts over 5 second epochs using the software’s default filter (ω_0_ = 0.025 Hz, ω_1_ = 2.5 Hz). Next, raw “.csv” files were processed using the GGIR software (version 1.6–0, https://cran.r-project.org/web/packages/GGIR/)^24,31^. The processing methods of GGIR involved: (1) Auto-calibration of the data according to the local gravity^32^; (2) calculation of ENMO, LFENMO and MAD and inclusion of the previously obtained counts over 5 seconds epochs (Table 1) to participant datasets to follow the same non-wear time calculation and processing decisions than the rest of the acceleration metrics; (3) detection of the non-wear time based on the raw acceleration from the three axes using a validated algorithm^24^, briefly, each 15-min block was classified as non-wear time if the standard deviation of 2 out of the 3 axes was lower than 13 m*g* during the surrounding 60-min moving window or if the value range for 2 out of the 3 axes was lower than 50 m*g*; (4) detection of sustained abnormal high accelerations, i.e., higher than 5.5 g; (5) imputation of detected non-wear time and abnormal high accelerations by means of the acceleration for the rest of the recording period during the same time interval than the affected periods; and 6) separation of waking and sleeping hours using a validated algorithm on the non-dominant wrist data and guided by logged timestamps by participants^14^. Logged times were 9 min (95% confidence intervals [CI_95%_]: −6 to 25 min) earlier and 17 min (CI_95%_: 2 to 32 min) later than accelerometer detected times for sleep onset and wake-up times, respectively. Finally, waking and sleeping hours detected from the non-dominant wrist were applied to the hip and dominant wrist measurements of each participant. All participants with at least 4 days with ≥16 hours wearing accelerometers were included in the analyses.

### Data analysis

Sedentary time and time spent in each PA intensity (i.e., light, moderate and vigorous PA) were estimated from the ENMO metric from the dominant wrist and non-dominant wrist-worn accelerometer data. Hildebrand *et al*.’s^8,11^ ENMO cut points developed for the non-dominant wrist were applied to the dominant wrist and non-dominant wrist data. Additionally, we calculated the same variables using the validated cut points incremented by 5 and 10 m*g*^8,11^ for the dominant wrist data only. Daily means of the acceleration metrics for 24 hours, waking hours and sleeping hours, as well as estimations of time spent in sedentary behaviors and PA intensity levels from the wrist-worn accelerometers were included in the analyses.

### Statistics

Descriptive statistics were calculated as means and standard deviations. We used linear regressions to study the associations between the different acceleration metrics (i.e., ENMO, LFENMO, MAD and counts) calculated from the same and different body attachment sites (i.e., right hip, dominant wrist and non-dominant wrist) (i.e., aim 1).

In order to study whether acceleration metrics identify a different movement pattern over the day (i.e., aim 2), we plotted 30-min averages of acceleration metrics across body attachment sites. As each acceleration metric has a different unit of measurement, we used z-scores when different acceleration metrics appeared in the same plot. Furthermore, we performed a curve analysis using statistical parametric mapping (SPM)^33^ to compare accelerations from dominant and non-dominant wrists throughout the day. Acceleration data over the day were depicted as curves. These acceleration curves produced throughout the day are highly variable between individuals due to several factors (e.g., lifestyle, working schedule). To minimize this high variability and allow for a comparison of the curves, we sorted accelerations produced every day per participant in an increasing order. Therefore, all of the curves start with the periods of the day when activity was low (e.g., sleep, sedentary activities…) and finish with the periods of the day with the highest intensity activities, independently of the moment of the day when they occurred. T-tests were used to determine significant differences between the curves for dominant and non-dominant wrists. SPM involved 4 steps to compute the t-test analysis: (1) computing the value of a test statistic at each point in the normalized time series; (2) estimating temporal smoothness on the basis of the average temporal gradient; (3) an equally smooth random process is performed to compute the value of the test statistics above which only α = 5% of the data would be expected to reach; (4) computing the probability that specific suprathreshold regions could have resulted from an equivalently smooth random process.

Finally, the Lin’s concordance correlation coefficient (LCCC), two sample t-tests and mean absolute percent error (MAPE) were used to study the agreement between sedentary time and time spent in each PA intensity derived from non-dominant wrist with validated cut points (reference) and all cut points used for dominant wrist (see *Data processing* section) (i.e., aim 3).

Cut-point selection was made following these criteria: (1) closest vigorous PA estimation if any; (2) closest moderate PA estimation if any (the upper threshold is already defined in step 1); (3) closest light PA estimation if any (the upper threshold is already defined in step 2). When two or more thresholds provided similar results, we tried to respect the original distance between the previously-established thresholds for non-dominant wrist^8,11^. The closest thresholds were selected based on mean differences, LCCC and MAPE. Then, the selected cut points were cross-validated using data from the cut-point cross-validation sample. All analyses were performed in R v.3.4.1 (https://cran.r-project.org/). The significance level was set at p < 0.05.

## Results

### Descriptive characteristics of participants

Out of the 45 participants from the cut-point calibration sample, three were excluded from the analyses for either not having accumulated enough wear time (n = 2) or having mis-placed the dominant wrist and non-dominant accelerometers for ≥1 day (n = 1). All of the cut-point cross-validation sample participants were included in the cross-validation analyses (i.e., aim 3). Descriptive characteristics of the included participants, as well as acceleration metric values for each body attachment site (i.e., right hip, dominant wrist and non-dominant wrists) across periods of the day (i.e., 24 hours, waking hours and sleeping hours) are presented in Table 2. Since wear time was high and practically the same for the right hip, dominant wrist and non-dominant wrist accelerometers, we did not delete unmatched non-wear time across accelerometers.

**Table 2:** Descriptive characteristics of participants.

	Cut-point calibration sample			Cut-point cross-validation sample		
	All (N = 42)	Men (N = 19)	Women (N = 23)	All (N = 36)	Men (N = 26)	Women (N = 10)
Age (years)	27.3 (5.3)	27.2 (5.9)	27.4 (4.9)	24.3 (1.9)	24.4 (2.3)	24.1 (1.1)
Weight (kg)	67.8 (12.1)	78.3 (8.5)	59.1 (6.3)	70.3 (14.2)	77.0 (13.1)	58.3 (5.1)
Height (cm)	171.0 (8.3)	178.0 (1.7)	165.1 (5.2)	172.3 (9.7)	177.0 (7.8)	163.8 (6.6)
BMI (kg/m^2^)	23.0 (2.6)	24.6 (1.7)	21.7 (2.5)	23.5 (3.6)	24.5 (3.8)	21.8 (2.5)
	**Cut-point calibration sample (N = 42)**			Cut-point cross-validation sample (N = 36)*		
**Acceleration metrics**	**24 hours**	**Waking hours**	**Sleeping hours**	**24 hours**	**Waking hours**	**Sleeping hours**
**Wear time (h/day)**						
Right hip	23.9 (0.3)	17.2 (0.7)	6.7 (0.7)	—	—	—
Dom. wrist	24.0 (0.2)	17.0 (0.7)	6.9 (0.6)	23.7 (1.3)	16.2 (1.7)	7.5 (1.8)
Non-dom. wrist	24.0 (0.2)	17.0 (0.7)	7.0 (0.6)	23.6 (1.4)	16.2 (1.4)	7.4 (1.5)
**ENMO (m*g*)**						
Right hip	16.0 (5.6)	21.4 (7.7)	2.4 (1.3)	—	—	—
Dom. wrist	33.9 (7.6)	46.5 (10.6)	3.0 (1.5)	31.7 (14.0)	43.1 (13.8)	3.5 (2.3)
Non-dom. wrist	31.3 (6.8)	43.1 (9.9)	3.2 (1.4)	29.9 (12.9)	40.7 (12.8)	4.4 (4.8)
**LFENMO (m*g*)**						
Right hip	12.1 (4.7)	16.0 (6.4)	2.1 (1.1)	—	—	—
Dom. wrist	26.4 (6.4)	36.1 (8.9)	2.5 (1.2)	—	—	—
Non-dom. wrist	24.9 (5.9)	34.3 (8.6)	2.6 (1.2)	—	—	—
**MAD (m*g*)**						
Right hip	24.4 (6.9)	33.4 (9.4)	1.5 (1.7)	—	—	—
Dom. wrist	48.4 (8.9)	67.0 (12.2)	2.8 (2.2)	—	—	—
Non-dom. wrist	44.2 (8.5)	61.4 (12.0)	2.9 (2.1)	—	—	—
**Counts/5 s**						
Right hip	41.4 (9.9)	56.3 (13.6)	3.6 (3.0)	—	—	—
Dom. wrist	176.9 (31.8)	242.6 (43.2)	14.6 (8.9)	—	—	—
Non-dom. wrist	164.9 (29.3)	226.7 (41.6)	15.0 (7.8)	—	—	—

Data are presented as mean (standard deviation)

*Cut-point cross-validation sample data was only used to cross-validated cut points for dominant wrist based on ENMO, so they did not wear hip-worn accelerometers and only ENMO was derived.

BMI: Body mass index; ENMO: Euclidean norm minus 1 *g* with negative values rounded to zero; LFENMO: Euclidean norm minus 1 *g* of the low-pass filtered raw accelerations with negative values rounded to zero; MAD: Mean amplitude deviation.

### Comparison of each acceleration metric across body attachment sites

**Figure 1:**
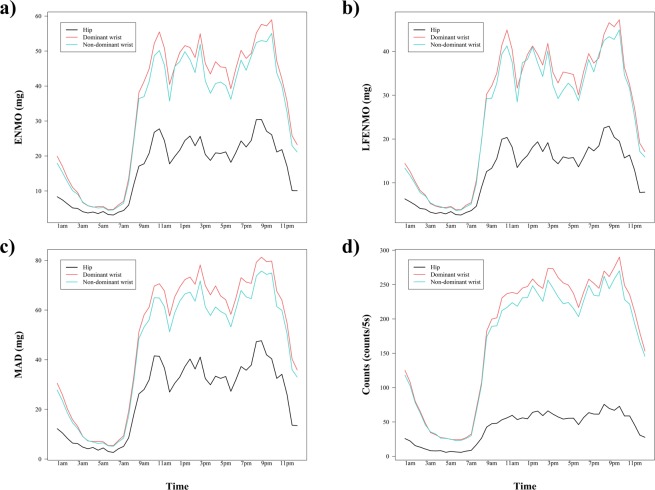
Means of ENMO (Panel a), LFENMO (Panel b), MAD (Panel c) and Counts (Panel d) over 30-min periods for the hip, dominant and non-dominant wrist. Each data point is the average for this time interval for all participants from the cut-point calibration sample (N = 42). ENMO: Euclidean norm minus 1 *g* with negative values rounded to zero; LFENMO: Euclidean norm minus 1 *g* of the low-pass filtered raw accelerations with negative values rounded to zero; MAD: Mean amplitude deviation.

**Figure 2:**
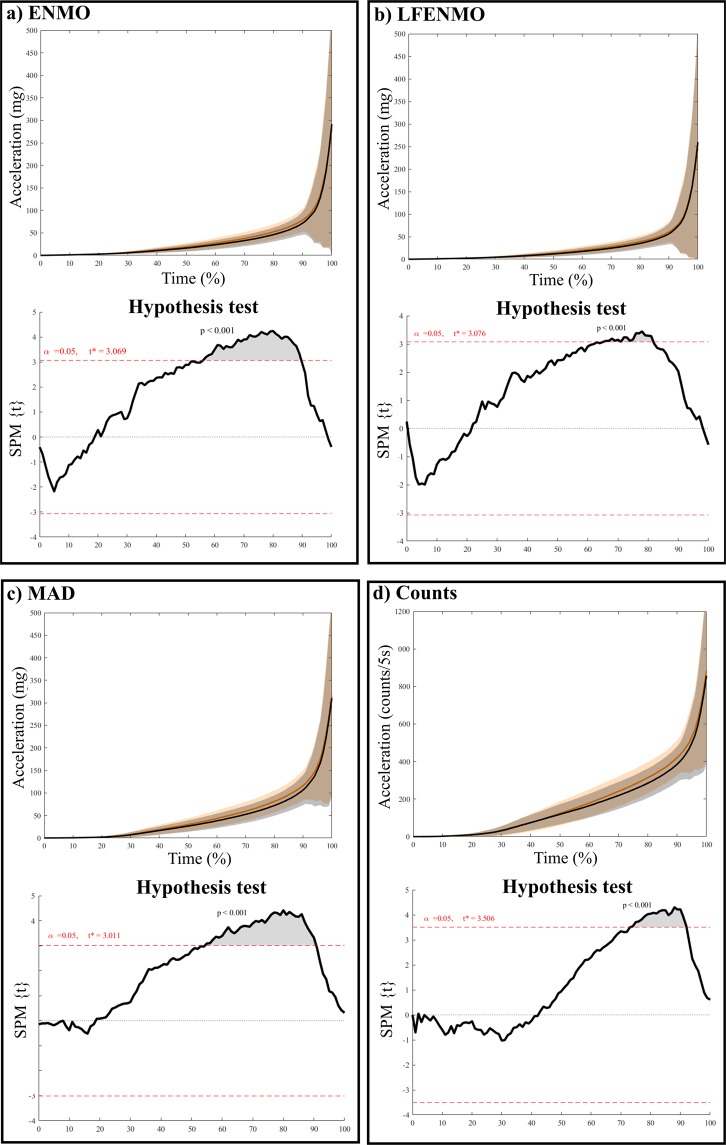
Comparison of means of ENMO (Panel a), LFENMO (Panel b), MAD (Panel c) and Counts (Panel d) sorted in an increasing order between dominant and non-dominant wrist. Each data point is the average for all participants from the cut-point calibration sample (N = 42). ENMO: Euclidean norm minus 1 *g* with negative values rounded to zero; LFENMO: Euclidean norm minus 1 *g* of the low-pass filtered raw accelerations with negative values rounded to zero; MAD: Mean amplitude deviation.

Table 3 presents the shared variances for every acceleration metric across different body attachment sites, i.e., hip, dominant wrist and non-dominant wrist. Overall, shared variance between wrists (r^2^ between 0.56 [CI_95%_: 0.33–0.74] and 0.94 [CI_95%_: 0.89–0.97]) was higher than shared variance between any of the wrists and the hip (r^2^ between 0.21 [CI_95%_: 0.03–0.45] and 0.88 [CI_95%_: 0.78–0.93]) for all the metrics analyzed. Figure 1 shows that acceleration values (for all of the metrics) for the wrists are higher than for the hip, with the highest values reached in the dominant wrist. Although the PA pattern seemed to be concordant for dominant wrist and non-dominant wrists, SPM analysis showed significant differences (p < 0.001) between the 50^th^ and the 90^th^ percentile of the accelerations produced (when accelerations start to increase, indicating periods of PA, see Fig. 2). In regards to the PA pattern identified from hip, besides recording lower values, peaks of activity were not totally concordant with those identified by the wrists.

**Table 3:** Shared variance (r^2^) for each acceleration metric across different body attachment sites (i.e., hip, dominant and non-dominant wrists).

	ENMO	LFENMO	MAD	Counts
**24 hours**				
Right hip vs. Dominant wrist	0.37**	0.28*	0.68**	0.40**
Right hip vs. Non-dominant wrist	0.34**	0.25*	0.77**	0.43**
Dominant vs. Non-dominant wrist	0.79**	0.78**	0.86**	0.71**
**Waking hours**				
Right hip vs. Dominant wrist	0.37**	0.31**	0.66**	0.38**
Right hip vs. Non-dominant wrist	0.35**	0.28*	0.75**	0.42**
Dominant vs. Non-dominant wrist	0.79**	0.79**	0.86**	0.70**
**Sleeping hours**				
Right hip vs. Dominant wrist	0.37**	0.21*	0.88**	0.75**
Right hip vs. Non-dominant wrist	0.39**	0.27*	0.88**	0.69**
Dominant vs. Non-dominant wrist	0.67**	0.56**	0.94**	0.92**

ENMO: Euclidean norm minus 1 *g* with negative values rounded to zero; LFENMO: Euclidean norm minus 1 *g* of the low-pass filtered raw accelerations with negative values rounded to zero; MAD: Mean amplitude deviation.

*p < 0.01

**p < 0.001.

### Comparison of acceleration metrics derived from the same body attachment site

**Figure 3:**
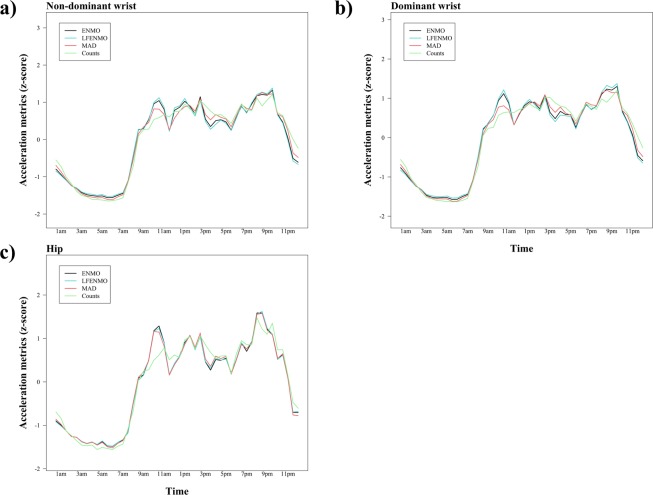
Means of ENMO, LFENMO, MAD and Counts, over 30-min periods for non-dominant wrist (Panel a), dominant wrist (Panel b) and hip (Panel c). Each data point is the average for this time interval for all participants from the cut-point calibration sample (N = 42). ENMO: Euclidean norm minus 1 *g* with negative values rounded to zero; LFENMO: Euclidean norm minus 1 *g* of the low-pass filtered raw accelerations with negative values rounded to zero; MAD: Mean amplitude deviation.

Table 4 shows the shared variances (r^2^) between pair of acceleration metrics derived from the same body attachment site and period of the day (i.e., 24 hours, waking and sleeping hours). ENMO and LFENMO were the metrics which presented the highest shared variances among all of the metrics included (r^2^ ranged from 0.95 [CI_95%_: 0.90–0.97] to 0.97 [CI_95%_: 0.93–0.98] for all locations and moments of the day). The lowest shared variances were found between LFENMO and Counts (r^2^ = 0.19 [CI_95%_: 0.02–0.42]), and for LFENMO and MAD during sleeping hours for the hip (r^2^ = 0.21 [CI_95%_: 0.03–0.45]). For the rest of the metrics, in general, they presented higher r^2^ values during waking hours (r^2^ between 0.38 [CI_95%_: 0.11–0.57] and 0.92 [CI_95%_: 0.85–0.96]) than during sleeping hours (r^2^ between 0.32 [CI_95%_: 0.10–0.55] and 0.79 [CI_95%_: 0.65–0.88]). A graphical comparison of all of the acceleration metrics for each body attachment site can be found in Fig. 3. While ENMO, LFENMO and MAD were describing almost the same movement pattern when derived from the same attachment site, counts were more discordant in some periods of the day (e.g., between 9 and 11 am, counts did not detect a peak of activity identified by the rest of the metrics in all of the placements, Fig. 3).

**Table 4:** Explained variance (r^2^) between different acceleration metrics derived from the same body attachment site (N = 42).

	ENMO vs. LFENMO	ENMO vs. MAD	ENMO vs. Counts	LFENMO vs. MAD	LFENMO vs. Counts	MAD vs. Counts
**24 hours**						
Right hip	0.97**	0.72**	0.46**	0.59**	0.34**	0.81**
Dominant wrist	0.96**	0.91**	0.55**	0.86**	0.51**	0.66**
Non-dominant wrist	0.97**	0.87**	0.49**	0.82**	0.47**	0.56**
**Waking hours**						
Right hip	0.97**	0.74**	0.48**	0.62**	0.38**	0.81**
Dominant wrist	0.97**	0.92**	0.54**	0.87**	0.51**	0.64**
Non-dominant wrist	0.97**	0.89**	0.52**	0.85**	0.51**	0.57**
**Sleeping hours**						
Right hip	0.95**	0.42**	0.32**	0.21**	0.19*	0.54**
Dominant wrist	0.97**	0.75**	0.44**	0.59**	0.37**	0.47**
Non-dominant wrist	0.97**	0.79**	0.42**	0.66**	0.37**	0.44**

ENMO: Euclidean norm minus 1 *g* with negative values rounded to zero; LFENMO: Euclidean norm minus 1 *g* of the low-pass filtered raw accelerations with negative values rounded to zero; MAD: Mean amplitude deviation.

*p < 0.01

**p < 0.001.

### Cut-point replication

As the shared variance between ENMO derived from the dominant and non-dominant wrist was fairly high during waking hours (r^2^ = 0.79 [CI_95%_: 0.65–0.88], Table 4), and the movement pattern over the day identified was very similar for both wrists (Fig. 1), we replicated the cut points validated by Hildebrand *et al*. in the non-dominant wrist^8,11^ using dominant wrist ENMO values (Table 5). After considering the criteria defined to select the new cut points, we found that the closest estimation between wrists was achieved with 50, 110 and 440 m*g* to classify sedentary time and light, moderate and vigorous PA intensities, respectively. Criteria were: (1) closest vigorous PA estimation if any, no threshold was selected since all of them were equally comparable; 2) closest moderate PA estimation if any, 110–430 mg, 110–435 mg and 110–440 mg provided similar results, so 110–440 mg was selected to respect the distance between previously-established cut points (i.e., 100–430 mg); (3) closest light PA estimation considering the upper threshold defined in step 2 (i.e., 110 mg). This selection of cut points was cross-validated using data from the wave 2; estimations were not significantly different between wrists for any of the intensities and showed high LCCCs (0.85–0.92) and low MAPEs (0.67–14.29%).

**Table 5:** Cut-point translation from previously proposed non-dominant wrist cut points for dominant wrist ENMO.

	Mean (SD) non-dominant wrist (min/day)	Mean (SD) dominant wrist (min/day)	LCCC	Mean diff. [95% CI] (min)^b^	MAPE
**Cut-points translation**					
*Sedentary time thresholds (mg)*					
45^a^	769 (69)	744 (71)	0.86	−25 [−55 to 6]	3.25%
**50**	—	**766 (69)**	**0.92**	**−3 [−33 to 27]**	**0.39%**
55	—	786 (67)	0.90	17 [−13 to 46]	2.21%
*Light PA thresholds (mg)*					
45–100^a^	147 (29)	161 (34)	0.84	14 [0 to 28]	9.52%
45–105	—	170 (37)	0.73	23 [9 to 37]	15.65%
45–110	—	179 (38)	0.63	31 [17 to 46]	21.09%
50–100	—	139 (30)	0.91	−8 [−21 to 5]	5.44%
50–105	—	148 (33)	0.93	1 [−12 to 15]	0.68%
**50–110**	—	**157 (34)**	**0.89**	**9 [−4 to 23]**	**6.12%**
55–100	—	120 (27)	0.62	−27 [−40 to −15]	18.37%
55–105	—	129 (29)	0.78	−19 [−31 to −6]	12.93%
55–110	—	137 (31)	0.88	−10 [−23 to 3]	6.80%
*Moderate PA thresholds (mg)*					
100–430^a^	104 (28)	120 (30)	0.72	16 [3 to 28]	15.38%
100–435	—	120 (30)	0.72	14 [0 to 28]	13.46%
100–440	—	120 (30)	0.72	16 [4 to 29]	15.38%
105–430	—	111 (28)	0.82	7 [−5 to 19]	6.73%
105–435	—	111 (28)	0.81	7 [−5 to 19]	6.73%
105–440	—	111 (28)	0.81	7 [−5 to 19]	6.73%
110–430	—	103 (26)	0.84	−1 [−13 to 10]	0.96%
110–435	—	103 (26)	0.84	−1 [−13 to 11]	0.96%
**110–440**	—	**103 (26)**	**0.84**	**−1 [−13 to 11]**	0.96%
*Vigorous PA thresholds (mg)*					
430^a^	9 (7)	9 (7)	0.95	0 [−3, 3]	0%
435	—	9 (7)	0.95	0 [−3, 3]	0%
**440**	—	**9 (7)**	**0.95**	**0 [−3, 3]**	0%
**Cross-validation**					
Sedentary time	750 (78)^c^	755 (94)^d^	0.85	5 [−36, 45]	0.67%
Light PA	134 (34)^c^	133 (36)^d^	0.89	−2 [−18, 15]	1.49%
Moderate PA	103 (44)^c^	97 (44)^d^	0.92	−6 [−26, 15]	5.82%
Vigorous PA	7 (7)^c^	7 (7)^d^	0.90	1 [−2, 4]	14.29%

Cut-point selection (values presented in bold) was made following these criteria: 1) closest vigorous PA estimation if any, no threshold is selected since all of them were equally comparable; 2) closest moderate PA estimation if any, 110–430 m*g*, 110–435 m*g* and 110–440 m*g* provided similar results, so 110–440 m*g* was selected to respect the distance between previously-established cut points (i.e., 100–430 m*g*); 3) closest light PA estimation considering the upper threshold defined in step 2 (i.e., 110 m*g*).

PA: Physical activity; LCCC: Lin’s concordance correlation coefficient; CI: Confidence interval; SD, standard deviation; MAPE, mean absolute percent error

Bold text indicates the cut-point selection based on the criteria defined above.

^a^Indicates original cut points validated in non-dominant wrist^8,11^.

^b^Dominant wrist *minus* non-dominant wrist.

^c^Derived with the original cut points validated in non-dominant wrist, i.e., 45/100/430^8,11^.

^d^Derived with the cut points proposed in the present study for dominant wrist, i.e., 50/110/440.

## Discussion

### Main findings

The main findings of this study were: (i) the dominant wrist showed systematically higher acceleration metric values than the non-dominant wrist, which were translated into different sedentary time and PA estimations when using the same cut points; (ii) dominant and non-dominant wrist based estimations of PA became comparable by modifying the validated cut points, which was confirmed in a cross-validation sample; (iii) non-dominant and dominant wrist acceleration metrics shared a higher proportion of variance than between the hip and either wrist, while MAD was the metric with the highest shared variances across body attachment sites; (iv) overall, the metrics ENMO, LFENMO and MAD shared higher proportions of variance than any of these metrics with counts, especially when the metrics were derived from the wrist-worn accelerometers; (v) the movement pattern identified throughout the day was visually equivalent for any given acceleration metric (e.g., ENMO) from the dominant wrist and non-dominant wrist, and was also similar when comparing either wrist with the hip and across acceleration metrics derived from the same body site (with the exception of counts). Altogether, these findings demonstrate the extent to which different factors related to data collection (e.g., anatomical wear location) and processing procedures (e.g. different accelerometer metrics) could modify the final PA, sedentary time and sleep estimations.

### Comparison of each acceleration metric across body sites

Comparisons between each metric derived from different body attachment sites add important knowledge to the field. Studies attaching accelerometers to the hip (e.g., WHS^34^, NHANES 2003–2004 or ISCOLE), to the dominant wrist (e.g., UK Biobank), and to the non-dominant wrist (e.g., NHANES 2012–2013, and Whitehall II Study^35^) could reach conflicting conclusions in regards to PA, sedentary time and/or sleep outcomes. This study quantifies these potential differences. Accordingly with previous evidence^18^, our findings show higher associations between the dominant and non-dominant wrists than between either wrist and the hip for all the metrics included. Comparison of acceleration metrics across the body attachment sites revealed higher shared variances for MAD compared with the rest of the metrics. Furthermore, comparisons between the dominant wrist vs. hip, and the non-dominant wrist vs. hip were similar for all of the metrics included, as occurred in a previous study in adults^20^. In regards to the movement pattern throughout the day, we observed almost identical patterns between the dominant and non-dominant wrists (with slightly lower values for the non-dominant wrist). To a lesser extent, hip movement pattern was similar to those from wrists. This suggests that the relationship between the dominant and non-dominant wrist accelerations is linear, which is also supported by a previous study in adults^18^. This implies that activities across the day should be captured similarly by all sites, especially between the dominant and non-dominant wrist.

### Comparison of acceleration metrics derived from the same body attachment site

Our findings show moderate to high associations between pairs of acceleration metrics derived from the same body attachment site. Likewise, previous findings by van Hees *et al*.^24^ reported moderate to high shared variances between ENMO and other acceleration metrics (r^2^ from 0.46 to 0.95) not included in the present study. Also in concordance with van Hees *et al*.’s study^24^ in adults, our findings show stronger associations between pairs of metrics with none or minimal filtering (ENMO, LFENMO and MAD) than the comparisons of any of these metrics with counts, which may be explained by the application of a frequency filter to the raw signal. We also found that the movement pattern identified throughout the day was visually similar for ENMO, LFENMO and MAD, while counts did not identify some peaks of movement detected by the rest of the metrics. The current study complements the study by van Hees *et al*.^24^ by using 24 hours of accelerometer recording for both the hip (only waking hours in the previous study) and both wrists, as well as including different acceleration metrics for comparison, i.e., LFENMO, MAD and ActiGraph’s counts^23,24,26,36^. Furthermore, pairwise comparisons of acceleration metrics showed better agreement during waking hours than during sleeping hours in all the body attachment sites. However, it is important to note that absolute values of all acceleration metrics are lower during sleeping hours, which could produce these lower shared variances even when the absolute differences are rather small (see descriptive values in Table 2). To the best of our knowledge, this is the first study providing this comparison stratified by waking and sleeping hours.

### Cut-points replication

The fact that there are only cut points available to assess PA and sedentary time from the non-dominant wrist makes their application to data from the dominant wrist controversial. Indeed, differences found between acceleration curves from the dominant and non-dominant wrists indicate the need to propose new cut points for the dominant wrist. Accordingly, we detected 25 min/day less of sedentary time, 14 min/day more of light PA, 16 min/day more of moderate PA and similar estimations of vigorous PA using the original cut points (i.e., for non-dominant wrist) in data collected from the dominant wrist. The linearity in the association between wrists (i.e., consistent movement pattern detected from both the dominant and non-dominant wrists) make it possible to adapt cut points developed for one of the wrists to the other by applying slightly different new cut points. This strategy has been used previously by Rowlands *et al*. to replicate hip-based moderate-to-vigorous PA cut points using non-dominant wrist data^37^. In the present study, we develop cut points for the dominant wrist using previously validated cut points for the non-dominant wrist as reference^8,11^; and then, we cross-validated these newly developed cut points in a different sample. Estimations from previously validated cut points on the non-dominant wrist and their translation to the dominant wrist were almost equal and highly correlated. The cut points we propose in this study for the dominant wrist could help to obtain equivalent and comparable estimations of PA between studies using the dominant wrist attachment with studies using the non-dominant wrist attachment.

This study complements existing information by using a long-term measurement (7 days) for these comparisons since previous studies used 1-day measurements^18,20^. Furthermore, our stratified analyses for waking and sleeping hours allow an understanding of how acceleration metrics agree or disagree for PA, sedentary time and sleep-related estimations. Notably, our 24-hour-based comparisons across body attachment sites and acceleration metrics are similar to waking-hour-based comparisons.

This study has practical implications for studies using the same acceleration metric, but attaching accelerometers to different body sites or vice versa. Furthermore, accelerometers are not only used to estimate PA and sedentary time, but also to assess sleep. Researchers focused on any of these behaviors can benefit from the comparisons presented in this study across acceleration metrics and body attachment sites during waking and sleeping hours, since these associations were different depending on the period of the day analyzed. Finally, this study provides information to quantify methodological discrepancies across studies, as it provides cut points to get similar PA estimations from dominant wrist and non-dominant wrist. We suggest these cut points are used to obtain comparable estimations across studies. It should be noted that differences found between sites and acceleration metrics do not constitute different associations between sedentary time, physical activity and/or sleep outcomes with health parameters. Whether associations with health parameters differ depending on data collection and processing decisions should be studied by future research.

The main limitation of this study is the lack of a criterion that would allow us to assess the accuracy of each acceleration metric in the measurement of PA and/or sleep. Likewise, the lack of an energy expenditure measure precludes us from deriving cut points for dominant wrist against a criterion. Thus, although our derived cut points may be of great value to identify PA from the dominant wrist, these cut points should be tested against an energy expenditure measure in future studies. Another limitation is the use of a convenience sample and all analyses were only carried out with data from one accelerometer brand (ActiGraph GT3X+), which could limit the generalization of our findings to other devices^38,39^, or even to different generations of the same brand^40^. Strengths of the present study were: 1) the fact that we used consistent data processing techniques with all the metrics (e.g., same calculation of non-wear time or waking and sleeping hours detection) which allow for a direct comparison between metrics and body attachment sites; and 2) the fact that our participants reached high wear times, allowing for a complete range of daily living accelerations.

## Conclusion

In conclusion, our findings suggest higher acceleration metric values in the dominant wrist compared with the non-dominant wrist. These differences could be attenuated by applying the new set of cut points provided in this manuscript. Furthermore, ENMO and LFENMO were the metrics that compared the best, and to some extent, they also showed good comparability with MAD for daily average values and for the movement pattern identified throughout the day. However, counts were demonstrated to be less comparable to the previously-mentioned metrics. Future studies should test which of these metrics and body locations are the best to accurately capture physical activity against a criterion (e.g., calorimetry).
